# Tree Morphologic Plasticity Explains Deviation from Metabolic Scaling Theory in Semi-Arid Conifer Forests, Southwestern USA

**DOI:** 10.1371/journal.pone.0157582

**Published:** 2016-07-08

**Authors:** Tyson L. Swetnam, Christopher D. O’Connor, Ann M. Lynch

**Affiliations:** 1 School of Natural Resources and the Environment, University of Arizona, Tucson, AZ, United States of America; 2 United States Forest Service, Rocky Mountain Research Station, Missoula, MT, United States of America; 3 Laboratory of Tree Ring Research, University of Arizona, Tucson, AZ, United States of America; Montana State University, UNITED STATES

## Abstract

A significant concern about Metabolic Scaling Theory (MST) in real forests relates to consistent differences between the values of power law scaling exponents of tree primary size measures used to estimate mass and those predicted by MST. Here we consider why observed scaling exponents for diameter and height relationships deviate from MST predictions across three semi-arid conifer forests in relation to: (1) tree condition and physical form, (2) the level of inter-tree competition (e.g. open vs closed stand structure), (3) increasing tree age, and (4) differences in site productivity. Scaling exponent values derived from non-linear least-squares regression for trees in excellent condition (n = 381) were above the MST prediction at the 95% confidence level, while the exponent for trees in good condition were no different than MST (n = 926). Trees that were in fair or poor condition, characterized as diseased, leaning, or sparsely crowned had exponent values below MST predictions (n = 2,058), as did recently dead standing trees (n = 375). Exponent value of the mean-tree model that disregarded tree condition (n = 3,740) was consistent with other studies that reject MST scaling. Ostensibly, as stand density and competition increase trees exhibited greater morphological plasticity whereby the majority had characteristically fair or poor growth forms. Fitting by least-squares regression biases the mean-tree model scaling exponent toward values that are below MST idealized predictions. For 368 trees from Arizona with known establishment dates, increasing age had no significant impact on expected scaling. We further suggest height to diameter ratios below MST relate to vertical truncation caused by limitation in plant water availability. Even with environmentally imposed height limitation, proportionality between height and diameter scaling exponents were consistent with the predictions of MST.

## Introduction

Estimating the mass (M) of an individual tree from primary size measures, i.e. bole radii or diameter (D) and height (H, equivalent to path length of the longest branch), relies upon assumptions about the conservation of allometric relationships within each tree relative to its current health condition, physical form, and age. Across stands of trees, competition for resources and differences in potential productivity caused by topographic variation affect tree growth and physical responses, which are outwardly expressed via intraspecific allometry. Variation in allometry, within individuals and amongst stands of trees, impacts the quantity of tree-to-forest level biomass across a space-filled volume (in three dimensions) or area (in two dimensions). Such responses have major implications for biomass models based on remotely sensed measures, e.g. aerial lidar-derived forest canopy height profiles [1]. Metabolic Scaling Theory (MST) [2–6] predicts rates of change between primary size measures across size classes (the so-called ‘allometric covariation’) [7–11]. MST also predicts a space-filled density-dependent stand of trees at resource and demographic steady state to have a rank size-frequency distribution which exhibits a power law as an emergent feature [2,3,6,10–12]. Invaluably, MST is mechanistically grounded in open-system thermodynamics [4,13–15] which offers a framework for testable hypotheses. Currently, MST does not account for morphologic variations that result from life history, responses to physical damage, or to exogenous disturbance. Lines et al. [16] reported allometric relationships of H:D ratios, as well as canopy diameter, to vary predictably with environmental factors across a country-wide forest inventory. Duncanson et al. [17] found considerable variability in H:D relationships in 125,395 US Forest Inventory and Analysis (FIA) plots, suggesting that future extensions of MST should include demographic dynamics and recruitment factors to explain allometric variation. While a series of studies in tropical and temperate forests report that natural forests’ rank-size frequency distributions follow exponential distributions [18–22], advocates for MST have attempted to explain why covariation exists amongst primary size measures that deviate from predicted quarter power (¼ power-law) scaling [10–12]. It has also been shown that mean-tree size models of biomass exhibit increasing bias due to Jensen’s inequality [23], which in some cases may explain why MST predictions do not hold in real forests [24–26]. Still, uncertainty remains as to where the deviation from power-law scaling comes from. Here we examined how traits related to tree health and departure from physically ideal growth form leads to deviation in the exponents of MST-predicted allometric models.

### MST predictions

Besides bole radius (r) and diameter (D) the other primary size measure for estimating the aboveground volume of a tree, and thus its biomass, is branch path length equivalent to total height (H). MST predicts that when bole radius is the independent variable of height: *h* ∝ *r*^*α*^ [Eq. 1], the value of *α* for the idealized H:D case is *α* = 2/3 [6]. MST also makes predictions about the distribution of mass relative to radii where *m* ∝ *r*^8/3^ [Eq. 2] (see S1 File for details). Most allometric models use the logarithmic form: *ln*(*h*) = α *ln*(*r*) = *β* [Eq. 3][27–30], which is equivalent to a power law: *h* = *βr*^*α*^ [Eq. 4]. Importantly, most studies report an α [Eq. 2] which is consistently less than the 8/3 predicted by MST [16–31]. We did not explicitly test biomass in this study, however variation in the exponent for Eq. 1 should also apply to the allometry of biomass in Eq. 2 [6,17].

### Objectives

Reported scaling exponents consistently below MST-predicted values in the literature [16–31] spurred our interest in conducting these analyses. Our objective was to determine whether the allometric covariation amongst primary size measures which results in deviation from MST can be associated with either (a) endogenous or (b) exogenous factors which we had recorded in our own data. Historically, semi-arid forests only exhibit density dependent self-thinning in the most productive sites absent of disturbance. Past disturbances provided us with a wide range of structural conditions across environmental factors [32,33], from which we could test our simple hypotheses. Our null hypothesis was that the least-squares estimated exponent $\hat{\alpha }$ (Eqs. 2–4) would not be significantly different from MST regardless of individual condition or age (endogenous forcing), inter-tree competition or site productivity (exogenous forcing). Our first and second alternate hypotheses were that $\hat{\alpha }$ would be negatively, or positively, influenced by each endogenous or exogenous forcing, respectively.

For the endogenous factors we evaluated individual trees by health condition class and age. We evaluated condition class as a way of diagnosing which trees are successful competitors for limiting resources in a stand. We evaluated age to determine whether trees exhibit a greater deviation from expected scaling exponents as they get older. Exogenous factors included a range of stand densities and a gradient in net primary productivity (NPP), as estimated by ‘effective energy to mass transfer’ (EEMT), a thermodynamic model of available free energy that incorporates positive air and soil temperatures, annual precipitation, and vapor pressure deficit derived from observational data in a generalized form that can be continuously predicted across a landscape [34,35].

## Methods

To test our hypotheses, we used datasets from mixed conifer forests in two Madrean Archipelago Sky Island mountain ranges in Arizona [36–40], and one Southern Rocky Mountains forest in New Mexico [41–44]. All sites share similar climate and nearly identical forest types and species, but have different geologies (Table 1), see S2 File for further details. The individual tree data are available via the USFS Research Data Archive: http://dx.doi.org/10.2737/RDS-2016-0015.

**Table 1:** Location, climate, geology, and generalized forest types (Ponderosa Pine = PP, Mixed-Conifer = MC, White-fir dominated = WF, Spruce and Fir = SF, and Aspen Disclimax = AD) of the three study areas (see S2 File for a cross-walk of forest type descriptions). Weather data are from the Western Regional Climate Center [4,5] and Liu et al. [46]; forest type details are given the S2 File.

Site	N Lat.,W Long.	Elevation(m amsl)	MAT(C°)	MAP(mm yr^-1^)	Forest type
**Santa Catalina**	32.4°, -110.7°	2100–2,700	10°– 12°	420–940	PP, WF, MC
**Pinaleño**	32.7°, -109.9°	2300–3,267	5°– 12°	480–850	PP, WF, MC, SF, AD
**Jemez**	35.8°, -106.5°	2,234–3,431	3°– 9°	476–850	PP, WF, MC, SF, AD

### Study areas

**Fig 1:**
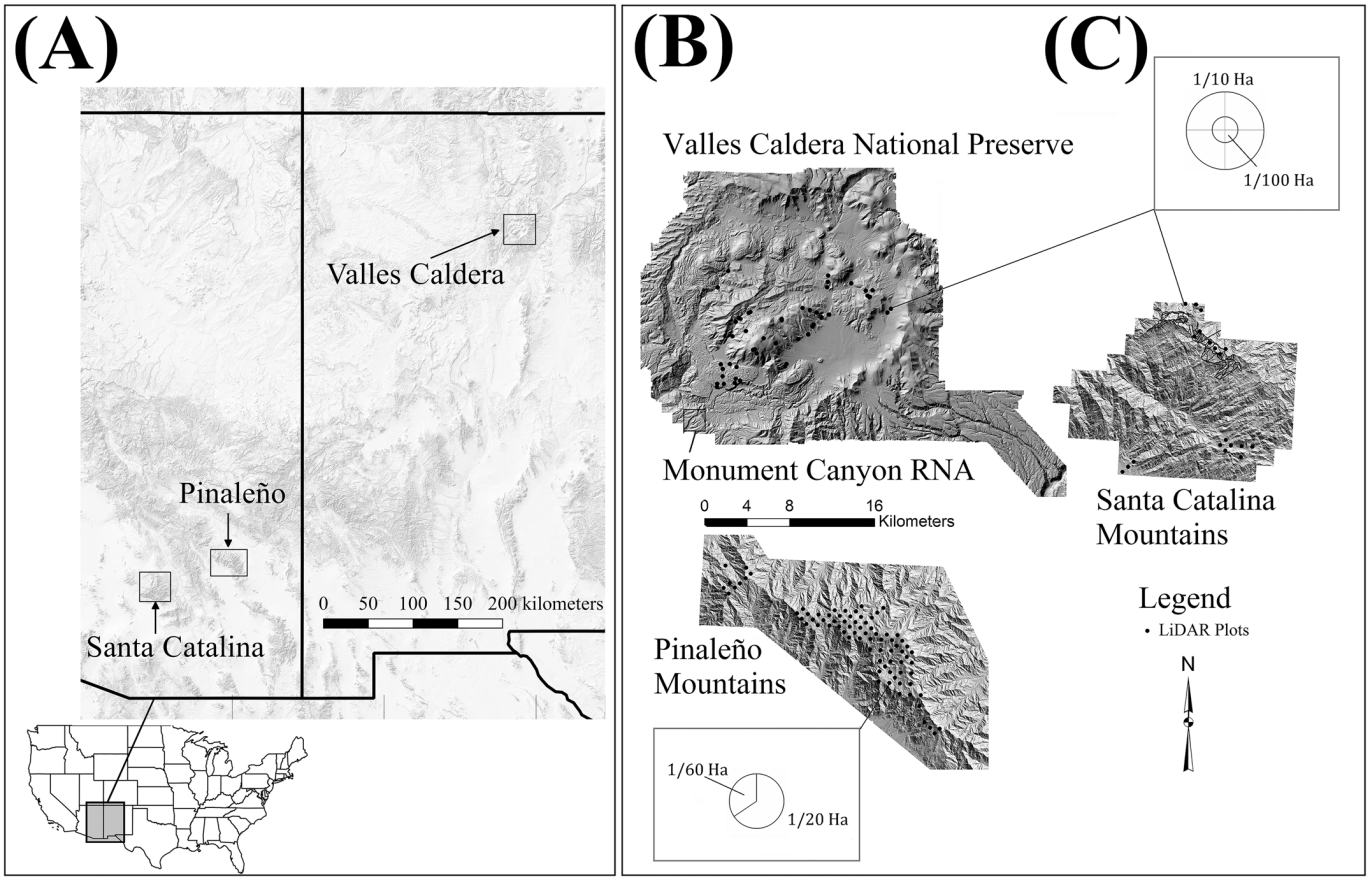
Study Area locations and plot design. (A) Location of the three study areas, (B) Surface models showing topographic variability and plot location, and (C) sampling plot layouts. Shaded relief in left panel via US Geological Survey, The National Map.

The Santa Catalina and Pinaleño Mountains are characteristic of basin and range topography, exhibiting complexes of steeply sided canyons at lower elevations with relatively gentle high elevation uplands. The Santa Catalina are located north of Tucson, Arizona, at 32.4° N, 110.7° W (Fig 1). The Pinaleños are located southwest of Safford, Arizona, at 32.7 N°, 109.9 W° (Fig 1). The Pinaleños are taller than the Santa Catalinas, rising to 3,267 meters (m) above mean sea level (amsl) and exhibit a mixed-conifer and spruce-fir forest above 2,700 m amsl (Table 1). Climatically, the Santa Catalinas and Pinaleños are nearly identical arid to semi-arid systems with a winter rainy season from December to March and a summer monsoon from July to September (Table 1). The Jemez Mountains are west of Santa Fe, New Mexico, at 35.8° N, 106.5° W (Fig 1). Elevation ranges from 2,300 m amsl to 3,431 m amsl (Table 1). The Jemez are considered continental sub-humid [38] though they are slightly drier (<50mm yr^-1^) and cooler (<3°C) than the Pinaleño Mountains (Table 1).

### Vegetation

Common tree species to this study occur across the Madrean Sky Island Archipelago, Mogollon Plateau, and Southern Rocky Mountains [36–38,41,47]. Multi-species forests are common in the study areas. For simplicity we use a simple classification system [48] for our forest types. In S2 File we provide cross-walked descriptions of these forest types for the Southwestern USA with associated references.

Across all three study areas forest types are distributed along elevation gradients of temperature and precipitation (Table 1). Variability in temperature and precipitation in our study areas is driven by orographic lifting related to topography and atmospheric lapse rates. Low elevations are generally hotter and drier, i.e. ‘xeric’, and high elevations are wetter and cooler, i.e. ‘mesic’. Forests generally extend lower in elevation along northern aspects and along mesic riparian drainages than southern aspects of the same elevation [36–38].

### Monitoring plots

Permission to collect samples and observational data were granted by the United States Forest Service Coronado National Forest, or were collected by USFS staff, for the Santa Catalina and Pinaleño data sets, and a research permit was granted by the Valles Caldera National Preserve for the Jemez data set. Common tree observations made at all locations included: species, condition, DBH (measured 1.37 m above ground level), and maximum vertical height (m). Tree height was measured with a hypsometer (Laser Technologies Inc. Impulse 200, Pinaleño; and Nikon Forester 550, Santa Catalina and Valles Caldera). Plot center locations were determined with a Trimble GeoXH global positioning system (GPS) with root mean square error (RMSE) of ±0.3 m horizontal distance.

In the summers of 2008 and 2009 technicians permanently staked, tree-tagged, and measured 79, 0.05 ha circular plots located along a systematic grid with 500 m spacing in the Pinaleño Mountains (Fig 1) [39,40]. Trees with DBH > 19.5 centimeters (cm) were measured over the entire plot area; trees with DBH 19.5 cm or smaller were measured within a 0.017 ha inner plot equivalent to 1/3 total plot area. Up to three increment cores were collected from each measured tree stem at a coring height ≤ 20 cm. Plots were limited to the area above 2,300 m amsl (Fig 1). Rationale for the use of systematic gridded plots for dendrochronological studies are described in further detail in previous studies at these sites [49–51].

In the summer of 2010 and spring of 2012, thirteen 0.1 ha plots were measured along a 1 km grid in the Santa Catalina Mountains using a tree sampling protocol identical to the Pinaleño Mountains (Fig 1). Tree measurements were identical to those of the Pinaleño plots. Also, in summer 2010, technicians established and measured 42, 0.1 ha plots in the Valles Caldera National Preserve (Fig 1). Locations were selected from a random distribution of points within 200 m of existing roads. The plot design and sampling protocol was identical to that used for the Santa Catalina.

### Tree condition

We partitioned our data by the trait “tree condition”, based on the Field Sampled Vegetation (FSVeg, [52]) inventory technique (see S3 File). Tree condition was determined by evaluating: (1) leaf condition including the color of the needles or leaves, (2) the density of needle fascicles per branch and overall volume and proportion of live canopy, (3) the vertical straightness of the standing bole, (4) evidence of parasites, diseases and physical damage, and (5) condition of the apical meristem leader. Condition was qualitatively categorized into (1) ‘excellent’, (2) ‘good’, (3) ‘fair’, or (4) ‘poor’ based on these five criteria and their associate scores (see S3 File for details). For the evaluation of dead standing trees we included only trees with intact brown needles or fine branches with unbroken leader stems.

### Age determination

Increment cores were mounted and sanded until individual cell structure was observable following standard dendrochronological procedures [53,54]. All samples used in the analysis were cross-dated using a combination of visual pattern matching [55], skeleton plots [54], and statistical pattern matching [56,57]. Reference chronologies used for cross-dating the Pinaleño samples were collected by Grissino-Mayer et al. [58]. Pith dates were estimated on samples that did not include pith but had enough ring curvature to uses concentric ring pith locators [59]. Only trees with estimated pith 10 years or fewer from the innermost sampled ring were used in tree-age analyses.

### Allometric models

We based our null model for H:D scaling on the MST prediction from West et al. [6,7] where α = 2/3. The relationship between height and diameter result in an equivalent scaling for the estimated tree aboveground biomass (AGB), M (kg), which is based on an assumption of volume preservation from the ‘pipe-model’ [60,61] in which trees support their phytomass through size-variable capillary networks that preserve cross-sectional area as they branch. This relationship is supported by recent tree dissection work of several tree species from the American southwest including trees from the Santa Catalina Mountains [7–9]. For the pipe model, tree total cubic volume (*V*) [cm^3^] is considered proportionate to a cylinder: *V* ∝ *πr*^2^*h* [Eq. 5], where bole radius *r* and h is tree height [cm] [15,27]. Mass is given as: *M* = *V* * *ρ* [Eq. 6], where *M* is the AGB, and *ρ* is the mean specific gravity [$\frac{g}{cm^{3}}$] of each species [27,62]. The use of an additional taper term in Eq. 6 is also used in some examples [24] but was not considered here. Because we did not measure biomass directly in this study, our models of estimated biomass should be considered theoretical in practice (see S1 File for full details).

### Productivity measured by EEMT

Historical observations of the flora and fauna in the Santa Catalina and Pinaleño Mountains [36] led to the concept of ‘gradient ecology’ [37], linking temperature, precipitation, and solar irradiation to NPP, species distributions, and biodiversity dynamics [38]. More recently, spatial models of EEMT [34,35,63,64] that account for the effects of forests on soil production rates and ridge-to-valley distances were developed in these same mountain ranges and applied to the stability of plant functional types following a change in disturbance frequency [47]. The form of the model is: *EEMT*[*Mjm*^−2^*yr*^−1^] = *f*(*T*, *VPD*, *PPT*, *Rn*, *CO*2)[*Wm*^−2^], where *T* is temperature [K], *VPD* is vapor pressure deficit [Pa], *PPT* is precipitation [kg m^-2^ s^-1^], *R*_*n*_ is net solar radiation [W m^-2^], and CO_2_ is carbon dioxide [kg m^-2^ s^-1^] [64]. The components of EEMT are equivalent to the requirements of plants for conducting metabolic activity, e.g. sunlight, positive air temperatures, and the presence of liquid water, and make it a suitable model for evaluating potential NPP [63]. We used the 10 m EEMT spatial GIS models from Rasmussen et al. [34] and Pelletier et al. [63] which represent the summation of the average EEMT over an entire year [MJ m^-2^ yr^-1^] to categorize the potential plant available free energy at each observed plot location for all three study areas. We extracted the EEMT value of each georeferenced plot location in ArcGIS 10.1 [65]. Santa Catalina and Jemez EEMT layer data are online available from: http://criticalzone.org/catalina-jemez/data/, the Pinaleño EEMT are available on request from the corresponding author of Ref. 63.

### Distribution fitting

Model fitting used non-linear least-squares regression with a Trust-Region algorithm in Matlab 2014a [66] Curve Fitting Tool. The continuous data for H:D were fit to a power law (Eq. 4) for a general model as well as (1) five condition classes, (2) three linearly binned age classes, and (3) five linearly binned EEMT categories. We used binning because the size of the samples for individual plots or ages was too small to generate narrow confidence intervals. We report the estimated *α* as $\hat{\alpha }$, and its upper and lower 95% confidence intervals (*ci*) for Eq. 4. Estimating power law exponents using least-squares regression and reporting their 90% to 95% *ci* is a common technique used by others [17,17–31], despite its known biases [23].

## Results

Estimates of the H:D scaling exponents (Eq. 4) were based on tree health condition of 3,740 physically intact trees. A total of 368 pith-dated trees from the Pinaleños were used for the age analysis. In Tables A, B, and C in S1 File we include estimates of M:D and M:H general models by study area, forest type, and species.

### Tree condition

**Fig 2:**
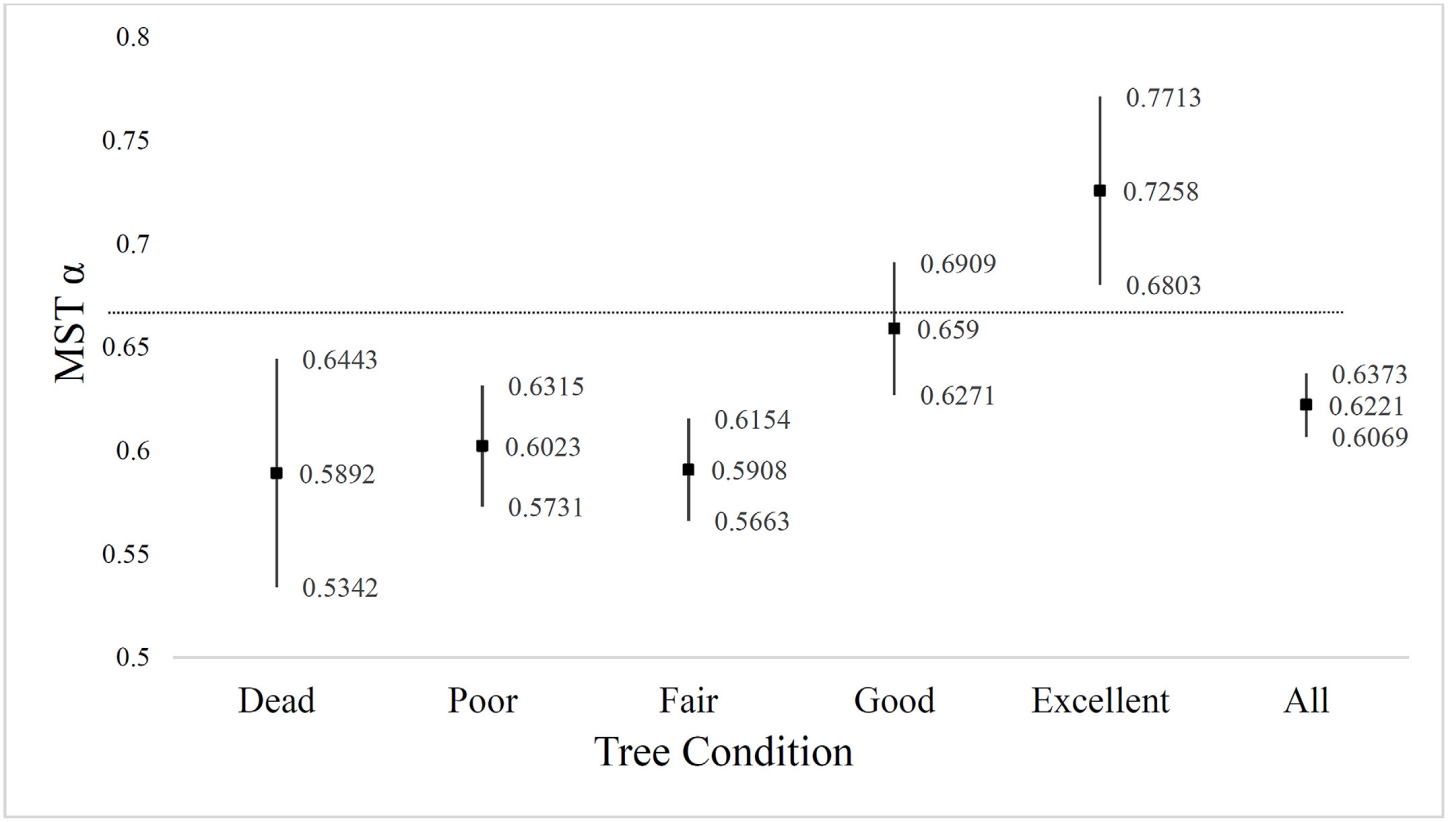
Scaling exponent values for Eq. 4 by tree condition classes. Least-squares regression of $h\;=\;\beta r^{\hat{\alpha }\;}$, estimated scaling exponent $\hat{\alpha }$ ± the 95% *ci* [Eq. 2, on the y-axis] (vertical black lines) tree condition [on the x-axis]. Trees with the most vigorous growth forms resulted in power law models with scaling exponents slightly above and not significantly different from the MST predicted 2/3 scaling (horizontal dashed black line). Trees in lower condition classes were significantly below MST-predicted scaling, as was the average of all trees.

We grouped trees into four live condition classes and a single dead class (Fig 2, Table 2). The scaling exponent $\hat{\alpha }$ (Eq. 4) of trees in excellent condition (n = 381) was significantly greater than the prediction of α = 2/3 predicted by MST (${\hat{\alpha }}_{2}\;=\;0.726\pm 0.046$). The scaling exponent of trees in good condition (n = 926) (${\hat{\alpha }}_{2}\;=\;0.659\pm 0.032$) was not significantly different than the MST prediction. In contrast, the scaling exponents of trees in fair condition (n = 923) (${\hat{\alpha }}_{2}\;=\;0.590\pm 0.025$), and poor condition (n = 1135) (${\hat{\alpha }}_{2}\;=\;0.602\pm 0.029$) were significantly less than MST predictions (Table 2). Scaling exponents of recently dead trees (n = 375) were also significantly less than the MST prediction (${\hat{\alpha }}_{2}\;=\;0.589\pm 0.055$) (Fig 2). The mean-tree model, regardless of condition, was significantly less than the MST prediction (${\hat{\alpha }}_{2}\;=\;0.622\pm 0.016$).

**Table 2:** Qualitative tree condition categories with least-squares regression: $h=\hat{\beta_{1}}r^{\hat{\alpha_{1r}}}$(Eq. 4); these models do not differentiate between species or location. The $\hat{\alpha_{1r}}\pm ci$ is graphically shown in Fig 2; ** denotes significantly greater than α_1r_ at 95% *ci*; * denotes significantly less than α_1r_ at 95% *ci*.

Condition	*n*	$\hat{\beta_{1}}\pm ci$	$\hat{\alpha_{1r}}\pm ci$	RMSE *m*	*R*^2^
**Excellent**	381	2.016 ± 0.269	0.726 ± 0.046**	3.52	0.711
**Good**	926	2.635 ± 0.328	0.659 ± 0.032	3.46	0.702
**Fair**	923	3.112 ± 0.222	0.591 ± 0.025*	3.14	0.733
**Poor**	1,135	2.732 ± 0.233	0.602 ± 0.029*	4.00	0.640
**Dead**	375	2.835 ± 0.532	0.589 ± 0.055*		
**All**	3,740	2.762 ± 0.136	0.622 ± 0.016*	3.65	0.689
**MST**	--	--	0.666	--	--

### Diameter, height, and age

**Fig 3:**
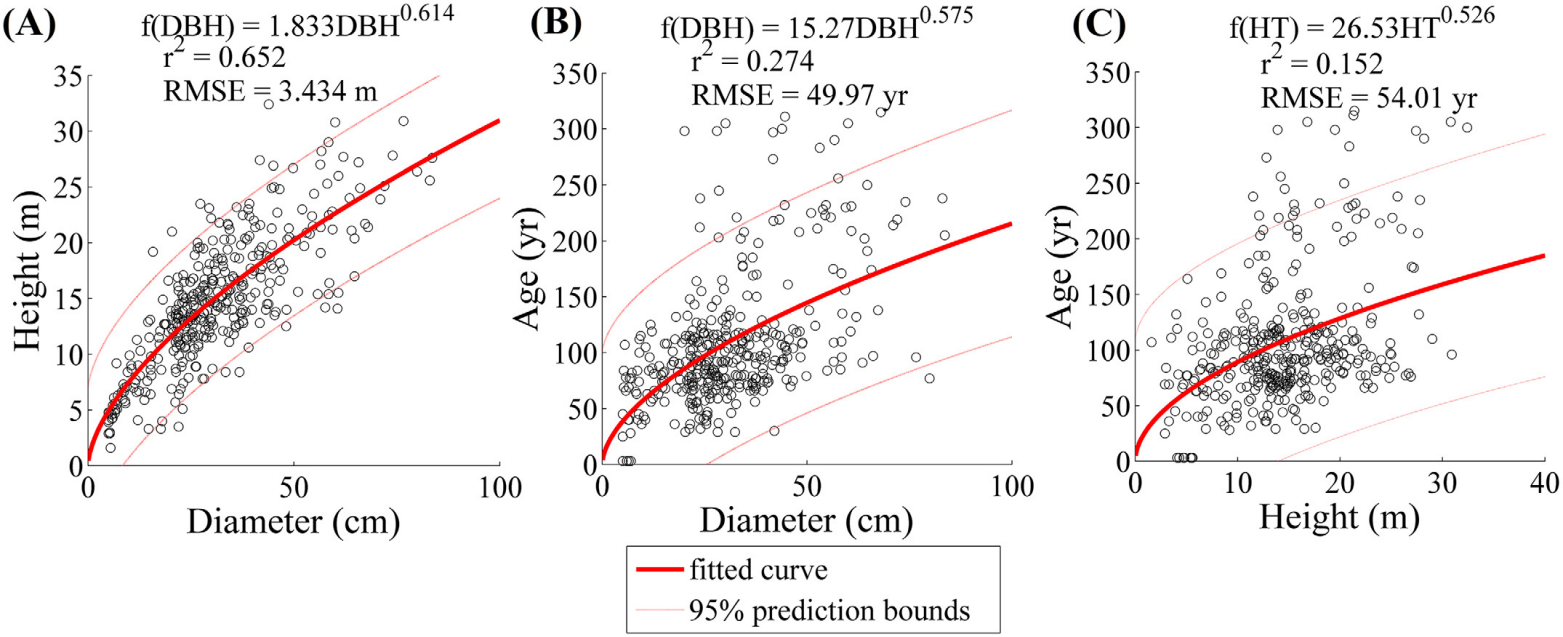
Least squares regressions of age, bole diameter, and height for dated trees from the Pinaleño (n = 368). (A) For diameter to height the correlation is fairly strong (r^2^ = 0.652); (B) diameter to age have a very weak correlation (r^2^ = 0.274) with wide heteroscedasticity; (C) similarly height has almost no correlation with increasing age (r^2^ = 0.152).

**Fig 4:**
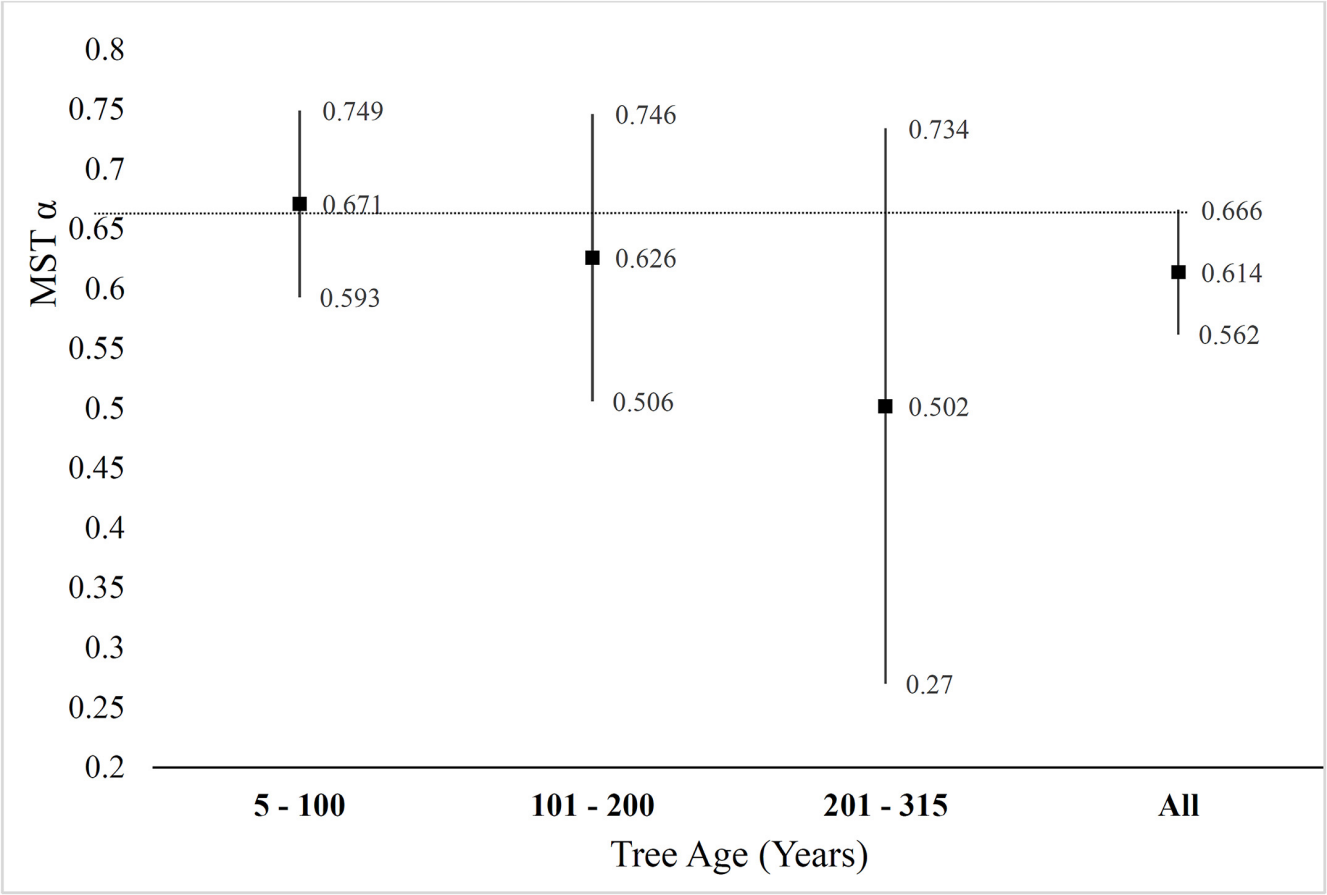
Estimated scaling exponent for Eq. 4 based increasing on tree age. The estimated scaling exponent $\hat{\alpha }$ ± 95% *ci* for diameter to height proportionality [Eq. 3, on the y-axis] of tree age. As trees increased in age there was a general decline in the estimated value for the proportionate scaling of $\hat{\alpha }$, though the difference was not significant at the 95% *ci* (vertical black lines).

In general, there was a weak relationship between tree size and age (Fig 3). The relationship between DBH and age (r^2^ = 0.274, RMSE = 49.97 years) was stronger than height and age (r^2^ = 0.152, RMSE = 52.01 years) but both distributions show characteristically poor fits (Fig 3) which exhibit wide heteroscedasticity. To compare the estimated scaling exponents of Eq. 2 by age, we grouped trees into three 100-year age classes. While there was a declining trend in the value of ${\hat{\alpha }}_{1r}$ for trees of increasing age, the difference in *α*_2_ was not significant at the 95% confidence level (Fig 4).

### Productivity gradient

**Fig 5:**
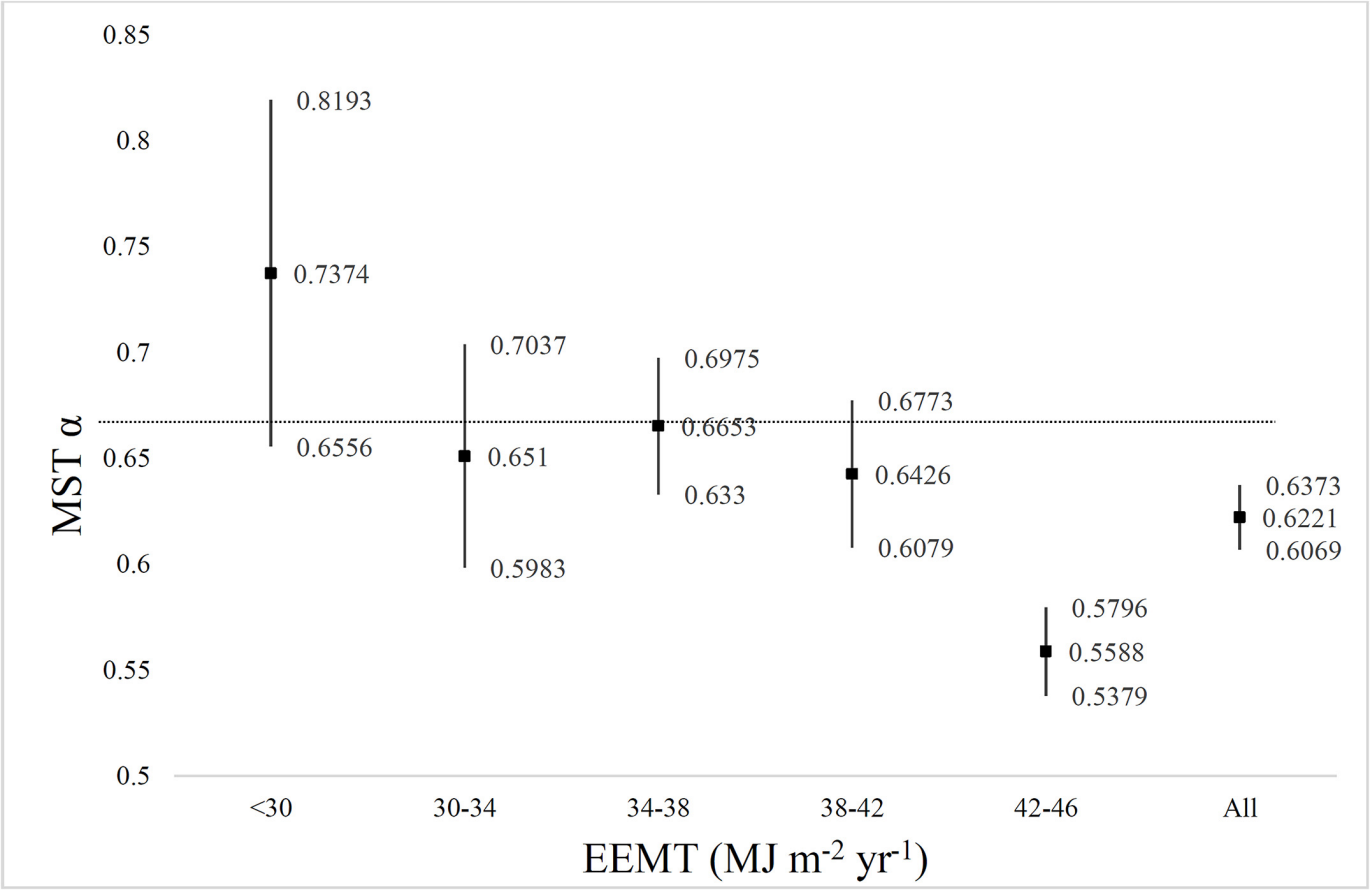
Estimated scaling exponent of Eq. 2 by EEMT. Least-squares regression by EEMT (MJ m^-2^ yr^-1^) [on the x-axis] versus the estimated scaling exponent $\hat{\alpha }$ ± 95% *ci* for diameter to height proportionality [Eq. 3, on the y-axis] (vertical black lines). There was no significant difference between the first four EEMT groups. There was a trend of declining $\hat{\alpha }$ as EEMT increased.

The largest trees by species in this study were Douglas-fir growing in mesic mixed-conifer/white-fir forest. The highest EEMT sites were the most productive and had the greatest biomass (S4 File). We grouped all plotted trees regardless of study area into five equally sized linear bins and assigned trees by plots to each bin. Binning was necessary because there are not enough trees in each plot to produce a reasonably narrow estimate of the scaling exponent values. As EEMT increased the $\hat{\alpha }$ value decreased across each successive group (Fig 5). The $\hat{\alpha }$ value was not significantly different from MST predictions for the first four bins, i.e. trees located in sites with EEMT <42 Mj m^-2^ yr^-1^. Only the last group (42–46 Mj m^-2^ yr^-1^), was significantly less than the MST prediction, as was the average of all trees for the entire study.

## Discussion

MST predictions were only met for trees with characteristically healthy growth forms (and in the case of the most robust canopy trees, exceeded); MST was rejected for trees in fair and poor health, as well as recently dead trees (Fig 2). Within the overall population, the number of ‘healthy’ trees (n = 1,307) are a little more than half the number of ‘unhealthy’ trees (n = 2,058). A non-linear least squares regression based on an entire population is therefore likely to skew the scaling exponent away from an MST prediction as seen in our data; for this reason, we suggest other forest studies which have rejected MST when developing models of M:D (see Tables A, B, C in S1 File) may do so because of the bias in their inventory related to mean-tree health.

Allometric scaling exponents of trees in our study were not significantly affected by increasing age, although there was a declining trend. Trees in lower density stands had a greater likelihood of exhibiting idealized MST scaling relationships, suggesting that trees with reduced competition are able to optimize resource uptake and allocation to wood production. Trees in denser stands that experienced direct competition for limiting resources deviated from ideal scaling, suggesting that competition has a direct negative effect on tree form.

Peak values in individual tree and total plot biomass increased along the increasing EEMT gradient (see S4 File). Stands with higher EEMT typically had a higher density of trees and a higher proportion of trees in below average health condition. This suggests that an increase in interspecific competition contributes to a greater number of individuals exhibiting poor growth form as they undergo density-dependent self-thinning. Plots with lower EEMT generally had lower biomass, conceivably a consequence of reduced water availability and the higher frequency, lower-severity fire regime common in pine-dominated stands of the region [40]. Trees in open stands tended to maximize their crown volume relative to trees in denser stands on higher EEMT sites.

The trees in this study had increasing divergence from MST for H:D with increasing age (Fig 3). One possible explanation involves limitations to maximum tree height [67–69], which are also in agreement with Darcy’s Law [70,71]. This concept of water-mediated vertical truncation is further supported by the finding that trees on well-drained ridge and hillslope sites are much shorter than trees in drainages and low topographic profile positions where water subsidy is greater [72]. Relative to temperate forests in Oregon and Washington with the same species, the trees from Arizona and New Mexico are little more than half the height of the largest members of their species [42–44,68]. Height limits to trees of the Southwestern USA more likely related to water availability, and not a mechanical limitation [69]. Anecdotally, trees that have met local critical thresholds in water movement up their meristem [71,73] could continue to accumulate mass as secondary growth on their branches [74]. Stephenson et al. [74] and Sillett et al. [75] report on an increasing rate of carbon accumulation in trees with increasing size and age. In studies of tree crown plasticity [76,77] trees subject to competition for light were not able to maximize crown volume or biomass.

### A more nuanced perspective of Metabolic Scaling Theory

Our findings suggest that when mean-tree condition diminishes, either from physical damage or disease, or from increased competition at higher stand densities, a non-linear least squares regression results in deviation from idealized MST scaling. This provides a possible explanation for the rejection of MST in numerous other forest ecology studies studying H:D and M:D relationships. We found that trees in the best physical condition exhibited MST-predicted scaling exponents. Intriguingly, the most vigorous trees with the largest crowns classified as being in ‘excellent’ condition had scaling exponents slightly greater than predicted by MST. Averaging the larger number of less healthy trees, essentially losing the long-term competition, with the relatively fewer healthy trees that are winning, results in forest-level scaling exponents that are lower than expected by idealized MST. We surmise that idealized scaling predicted by Eq. 4 [5,6,10] is conserved in healthy trees, but as trees become more stressed, either through competition, damage, or disease, their scaling is altered in the way Price et al. [11] demonstrated across a range of species. Allometric models developed from the mean-tree population using least-squares regression, calculated in a similar fashion as other studies [24,25,30,31] (Table A in S1 File), suggest when a large number of suppressed and stressed trees are present there is a reduction in the scaling exponent. This may explain why some MST predictions have been found to be inconclusive or unsupported by others [19,26]. A more nuanced interpretation of MST suggests that healthy trees, not subject to damage, disease or intense competition, are more likely to exhibit idealized MST scaling, while trees that are diseased, damaged, or losing the competition for light and water by self-thinning are more likely to scale below MST predictions.

## Conclusions

We found a common divergence from MST idealized exponents in mean-tree models to be explained by: (1) a majority of individuals exhibiting poor or fair growth form likely from increased resource competition and (2) a vertical height limitation that results in trees with lower than expected H:D proportionalities. Deviation in exponent values of height, radius and mass relationships, while significantly different from idealized MST, when averaged across all trees, were found to be consistent with MST for trees in good to excellent physical condition. Further, similar results across the study areas suggests our models are consistent enough to be applicable to other semi-arid forests in the Southwestern USA. Quantitative assessments of how competition, prevalence of disease, physical damage, mechanical stress, chronic moisture stress, or temperature extremes lead to covariation require further study. The difference between predicted and measured biomass for tall trees and old growth stands in vertically-truncated forests such as those of the Southwestern US also has important implications for calibrating aerial lidar-derived models [1], removing biases in allometric models [23, 78], particularly when large trees account for the majority of standing biomass [79].

While resolving specific mechanisms is beyond the scope of this study, we suggest future research should be directed at refining and testing possible mechanisms for deviation from idealized scaling, e.g. within trees the influence of scaling water flux with mass, as shown by Sperry et al. [80]; and outside of trees, the differences in soil depth and ground water availability which differentiate between specific drivers of tree height [72].

## Supporting Information

S1 File General forest biomass models and species biomass models. This file contains text, one figure and three tables labelled A, B, and C.
(PDF)

S2 File Vegetation classification schemes common for all three study areas. The suggested biomass model for each classification scheme: Plant Functional Type (PFT, Smith *et al*. 1993), Existing Vegetation Type (EVT, Comer *et al*. 2003, Brohman and Bryant 2005, Muldavin *et al*. 2006, LANDFIRE 2010), USFS mid-scale dominance types (Mellin *et al*. 2008), Plant associations (Stuever and Hayden 1997), Potential Natural Vegetation Type (PNVT, Nature Conservancy 2006, 2007), ReGap Analysis (Lowry *et al*. 2007, Prior-Magee *et al*. 2007), and Whittaker and Niering (1975)/Niering and Lowe (1984).
(PDF)

S3 File Tree Condition Codes. This file contains a workflow for classifying trees, a table of tree decay classes, and example plates of various tree species by class.
(PDF)

S4 File Pairwise comparisons of plot metrics for the Pinaleño and Valles Caldera study sites. Scatter plots for the Pinaleño (n = 79, gold circles) and Valles Caldera (n = 48, blue circles) inventory plots are classified by: Canopy Cover percentage (CC%), EEMT (MJ m^-2^ yr^-1^), biomass M (Mg ha^-1^), Basal Area (m^2^), trees per hectare (t / ha^-1^), and plot average Tree Health (excellent = 4, good = 3, fair = 2, and poor = 1). A least-squares trend line is included for each study area. The Santa Catalina data are not shown.
(PDF)
