# Supplementary material for: Tree Morphologic Plasticity Explains Deviation from Metabolic Scaling Theory in Semi-Arid Conifer Forests, Southwestern USA
Source: PLoS One. 2016 Jul 8;11(7):e0157582. doi: 10.1371/journal.pone.0157582 (PMC4938440; doi:10.1371/journal.pone.0157582)
Supplement: S1 File — This file contains text, one figure and three tables labelled A, B, and C. (PDF) [file pone.0157582.s001.pdf]

Supporting Information 1: General forest biomass models and species biomass models

MST and its related 'General Theory of Forest Structure' [2,3,6] predict the mass m_i, of an ith size tree, to be proportionate to its bole radius as: $m_i \propto r_i^{\alpha}$, where the scaling exponent $\alpha = \frac{2a+b}{a}$ [S1 Eq. 1], for an idealized case, a = 1/2 and b = 1/3 such that a and b are biologically meaningful parameters related to the space filling and volume preserving nature of organismal geometry [3,5,10,11]. For an ith bole radius tree the scalar measure of mass [kilograms, kg] based on radius [centimeters, cm] (from Eq. 1) is: $m_i = \beta_1 r_i^{\alpha_{1r}}$ [Eq. 2] where β_1 is a normalization constant, and $\alpha_{1r} = 8/3$ [6]. We did not measure mass directly in our study, so we have limited our reporting of the biomass models to this supplemental; these models are not intended to be used for any other applications without further validation.

In addition to examining the allometry of bole radius and mass (Eq. 1) we also evaluated the expected scaling exponent of tree height h_i (equivalent to bole length) as the independent variable to mass $m_i \propto h_i^{\alpha}$, $\alpha = \frac{2a+b}{b}$ [S1 Eq. 2], similar to SI Eq. 1 but the denominator is given as b rather than a [6]. Further, for a kth sized tree the scalar measure of mass based on height (m) is: $m_k = \beta_2 h_k^{\alpha_{1h}}$ [S1 Eq. 3] where β_2 is a normalization constant and $\alpha_{1h} = 4$.

The $\hat{\alpha}_{1r}$ [S1 Eq. 1] for the mean-tree model derived using the pipe-model technique for all trees in this study ($\hat{\alpha}_{1r} = 2.47 \pm 0.02$, $p \leq 0.05$) (S1 Table 1) was significantly less than the MST prediction of 8/3. Within the five forest types, $\hat{\alpha}_{1r}$ ranged from $2.358 \pm 0.038 < \hat{\alpha}_{1r} < 2.640 \pm 0.037$, $p \leq 0.05$ (S1 Table 2). Individual species $\hat{\alpha}_{1r}$ ranged from $2.22 \pm 0.05 < \hat{\alpha}_1 < 2.65 \pm 0.04$, $p \leq 0.05$ (S1 Table 3). As an example of the marginal differences between species-level models (S1 Fig 1), the relationship between mass and diameter based on $\hat{\alpha}_{1r}$ [S1 Eq. 1] for *P.*

ponderosa in our study ($\hat{\alpha}_{1r} = 2.64 \pm 0.037$, $p \leq 0.05$) (S1 Table 3), was slightly different than that of *P. arizonica* (a subspecies of *P. ponderosa*) ($\hat{\alpha}_{1r} = 2.574 \pm 0.026$) in northwestern Mexico [31], but not for *P. ponderosa* from other regions of western North America [27]($\hat{\alpha}_{1r} = 2.647$) (S1 Table 1). All of the species observed models were slightly lower than the MST prediction of $\alpha = 8/3$. In the species-level mean-tree model, scaling exponents fit by least-squares regression were consistently below MST predictions, a rejection of our null hypothesis and in agreement with other studies [18,19,25,26].

Price and Enquist [10] showed that covariation amongst primary size measures with increasing size arises from variation in the geometry of the vascular network which for Eq. 1 is idealized at *a*=1/2 and *b*=1/3. If the value of *a* or *b* are altered, the value of α for Eqs. 1 or 3 will also change. As an anecdotal example, a change in the space filling potential along the vertical height axis, altering *b* from 1/3 to ¼ for $m_i \propto r_i^{\left(\frac{2a+b}{a}\right)}$(Eq. 1), results in a change for Eq. 2, where α_{1r} changes from $\alpha_{1r} = 2.66\bar{6}$ to $\alpha_{1r} = 2.5$. If we compare the proportionality change in the estimated $\hat{\alpha}$ from Eq. 2 (Table 2) for all individuals over the expected MST α: $\frac{\hat{\alpha}}{\alpha} = \frac{0.622}{0.66\bar{6}} = 0.934$, such that for Eq. 2 the covariation *c*, $c * \alpha$: 0.934 * 2.66$\bar{6}$ = 2.490. This value is closer to what has been reported by Chave et al. [24] ($\alpha_1 = 2.510$), Jenkins et al. [29]($\alpha_1 = 2.435$), and Návar [30]($\alpha_1 = 2.396$) (S1 Table 1). The resulting change in proportionality of either *a* or *b* for population-averaged parameters that include both healthy and dying trees results in lower than expected scaling values, in particular when binning and least-squares regression are used [White et al. 2008].

Anecdotally, despite their truncated height, southwestern trees may continue to incrementally increase their bole diameter while adding mass onto their branches (S3 Plate 1). Failure to grow taller with increasing age results in larger diameter trees relative to a predicted height, resulting

in one possible source of covariation in the allometry for Eq. 1 and Eq. 3. For example, for $M \propto r^{\alpha_1}$ and $M \propto h^{\alpha_2}$ the observed scaling was: $\alpha_1 = 2.470 \pm 0.019$ and $\alpha_2 = 3.622 \pm 0.081$ (S1 Table 1); these values are proportional to each other with covariation *c*: $r^{c*8/3} \propto h^{c*4}$, where $c = 0.682$. While we cannot explicitly determine the idealized values of a or b parameters (Eq. 1) which result in α_1 and α_2, the change is proportional. Importantly, this covariation does not violate the MST prediction [10,11].

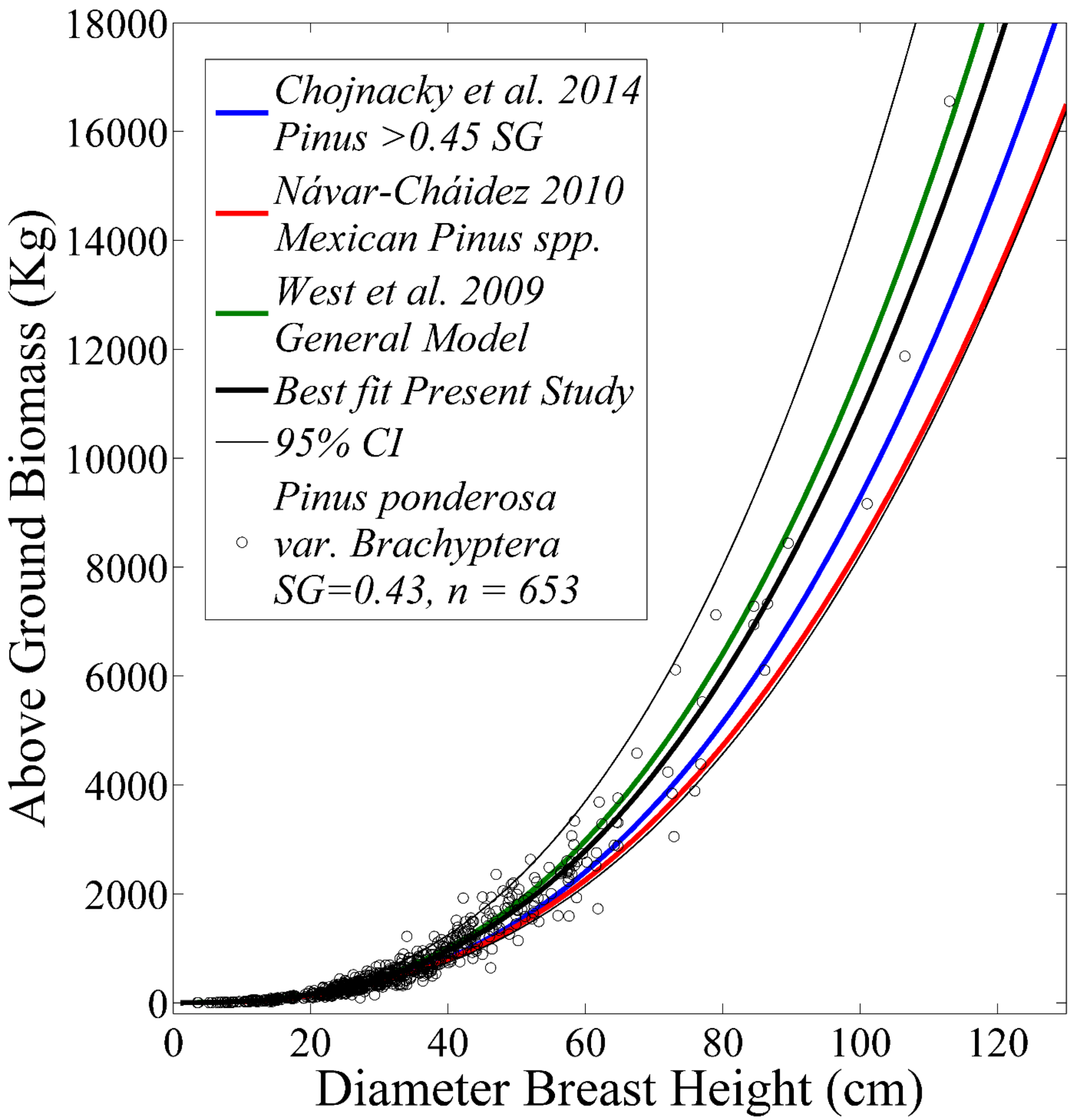

S1 Fig. Allometric model of biomass for southwestern ponderosa pine (*Pinus ponderosa var Brachyptera*). The non-linear models shown include: general *Pinus spp.* with specific gravity (SG) > 0.45 [27], Mexican pines including Arizona pine (*Pinus arizonica*), a subspecies of southwestern ponderosa pine [31]. The theoretical model of West *et al.* [6] uses a dummy β equal to our best fit model derived from least-squares regression of the data.

S1 Table A. General models of individual tree above ground biomass. General models of individual tree above ground biomass (M) (kg) which use the pipe model and primary size measures: radius (r) (1 cm linear bins) (Eq. 3) and vertical tree height (h) (1 cm linear bins) (Eq. 5), by study area, as well as other published biomass models; values not reported in the other publications are shown as '--'. §models graphically represented in Fig 3. ** denotes significantly greater at 95% ci, * denotes significantly less at 95% ci.

		$M = \beta_1 r^{\alpha_{1r}}$				$M = \beta_2 h^{\alpha_{1h}}$			
Present Study	N	$\beta_1 \pm ci$	$\alpha_{1r} \pm ci$	$RMSEkg$	R^2	$\beta_2 \pm ci$	$\alpha_2 \pm ci$	$RMSEkg$	R^2
Santa Catalina	300	0.148±0.036	2.599±0.056	335.3	0.977	0.007±0.007	3.94±0.28	927.6	0.822
Pinaleño	2,175	0.308±0.036	2.383±0.024 *	294.2	0.950	0.028±0.012	3.58±0.14 *	809.3	0.625
Valles Caldera	1,540	0.126±0.014	2.615±0.028 *	241.2	0.953	0.017±0.006	3.71±0.11 *	581.1	0.729
All Sites	3,740	0.222±0.018	2.470±0.019 *	307.5	0.9468	0.023±0.006	3.62±0.08 *	748.1	0.685
Other Studies									
Chojnacky *et al.* 2014§	--	0.0473	2.647	--	0.83	--	--	--	--
Jenkins *et al.* 2004	--	0.0793	2.435	--	--	--	--	--	--
Chave *et al.* 2005	--	--	2.500	--	--	--	--	--	--
Brown 1997	--	0.124	2.530	--	--	--	--	--	--
Návar 2009		0.123	2.396		0.91				
Návar-Cháidez 2010§	--	0.0597	2.574±0.026	--	0.86	--	--	--	--
West *et al.* 2009§	--	--	2.666	--	--	--	4	--	--

The estimated height scale exponent $\hat{\alpha}_{1h}$ [Eq. 4] for the general model was $\hat{\alpha}_{1h} = 3.622 \pm 0.081$, $p \leq 0.05$ (S1A Table 1). By forest type the $\hat{\alpha}_{1h}$ varied between $3.44 \pm 0.16 < \hat{\alpha}_{1h} < 3.95 \pm 0.19$, $p \leq 0.05$ (S1A Table 2), and by species, $2.46 \pm 0.31 < \hat{\alpha}_{1h} < 3.50 \pm 0.17$, $p \leq$

0.05 (S1 Table 3). Again, in most cases the value of $\hat{\alpha}_{1h}$ is an apparent rejection of the idealized MST parameter of height where $\alpha_{1h} = 4$ (Eq. 5).

S1 Table B. Forest type-level models for individual tree aboveground biomass. Models are based on either the observed radius (1 cm linear bins) or height (h) (1 m linear bins). ** denotes significantly greater at 95% ci, * denotes significantly less at 95% ci.

		$M = \beta_1 r^{\alpha_{1r}}$				$M = \beta_2 h^{\alpha_{1h}}$			
	n	$\beta_1 \pm ci$	$\alpha_{1r} \pm ci$	$RMSE$	R^2	$\beta_2 \pm ci$	$\alpha_2 \pm ci$	$RMSE$	R^2
**Pine Forest**	511	0.110±0.018	2.640±0.037	186.3	0.970	0.010±0.006	3.935±0.190	526.7	0.760
**Mixed-Conifer**	760	0.192±0.040	2.493±0.048*	363.5	0.921	0.027±0.013	3.605±0.143*	668.2	0.717
**White-fir**	1,177	0.236±0.034	2.448±0.033*	345.6	0.956	0.039±0.017	3.455±0.129*	779.8	0.755
**Spruce & Fir**	1,001	0.144±0.024	2.537±0.038*	195.6	0.952	0.036±0.019	3.443±0.164*	504.5	0.685
**Aspen**	378	0.314±0.050	2.358±0.038*	160.8	0.968	0.006±0.009	3.947±0.479	643.5	0.492

S1 Table C. Species-level models for individual tree aboveground biomass M (kg). Models by species based on radius using 1 cm linear bins. §The Ponderosa model is graphically shown in S1 Fig. ** denotes significantly greater at 95% ci, * denotes significantly less at 95% ci.

		$M = \beta_1 r^{\alpha_{1r}}$				$M = \beta_2 h^{\alpha_{1h}}$			
Species	n	$\beta_1 \pm SE$	$\alpha_1 \pm SE$	$RMSE$	R^2	$\beta_2 \pm SE$	$\alpha_2 \pm SE$	$RMSE$	R^2
Abies concolor	565	0.288±0.064	2.358±0.052*	215.2	0.944	0.054±0.033	3.36±0.17*	456.9	0.747
Abies lasciocarpa	247	0.128±0.036	2.571±0.076*	75.8	0.942	0.252±0.171	2.60±0.22*	147.8	0.735
Picea engelmanni	546	0.310±0.078	2.334±0.063*	1,740.0	0.923	0.195±0.094	2.84±0.16*	293.1	0.734
Pinus ponderosa§	653	0.108±0.016	2.651±0.035	251.6	0.965	0.036±0.020	3.50±0.17*	721.5	0.708
Pinus strobiformis	343	0.182±0.056	2.510±0.073*	282.2	0.935	0.450±0.336	2.70±0.23*	665.0	0.640
Pseudotsuga menziesii	730	0.362±0.076	2.371±0.047*	517.0	0.949	0.046±0.026	3.48±0.17*	1,123.0	0.752
Populus tremulodies	404	0.516±0.094	2.215±0.048*	98.3	0.939	0.357±0.341	2.46±0.31*	266.5	0.524
Quercus arizonica	55	0.214±0.322	2.323±0.410*	122.3	0.751	11.210±6.581	1.71±0.25*	139.1	0.677
Quercus gambelli	65	0.114±0.078	2.563±0.168	93.6	0.965	0.117±0.392	3.63±1.35	383.8	0.401
Robinia neomexicana	36	0.088±0.058	2.763±0.210	11.0	0.983	0.240±0.284	3.06±0.52*	25.2	0.907
