# Supplementary material for: Tree Morphologic Plasticity Explains Deviation from Metabolic Scaling Theory in Semi-Arid Conifer Forests, Southwestern USA
Source: PLoS One. 2016 Jul 8;11(7):e0157582. doi: 10.1371/journal.pone.0157582 (PMC4938440; doi:10.1371/journal.pone.0157582)
Supplement: S2 File — The suggested biomass model for each classification scheme: Plant Functional Type (PFT, Smith et al. 1993), Existing Vegetation Type (EVT, Comer et al. 2003, Brohman and Bryant 2005, Muldavin et al. 2006, LANDFIRE 2010), USFS mid-scale dominance types (Mellin et al. 2008), Plant associations (Stuever and Hayden 1997), Potential Natural Vegetation Type (PNVT, Nature Conservancy 2006, 2007), ReGap Analysis (Lowry et al. 2007, Prior-Magee et al. 2007), and Whittaker and Niering (1975)/Niering and Lowe (1984). (PDF) [file pone.0157582.s002.pdf]

Supporting Information 2: Forest Type and Species Descriptions

The five common PFTs in this study:

1) ***Ponderosa pine forest***: Sites were located from 2,100 to 2,750 m amsl with composition of >80% *Pinus ponderosa var. Brachyptera* (Engelm.) by volume (this includes the subspecies *Pinus arizonica* in the Santa Catalina)*;* other species included Southwestern white pine (variants in the literature are *P. strobiformis* (Engelm.), *P. reflexa* (Engelm.), or *P. flexilis* (James), here we do not differentiate between them, though there are minor taxonomical differences), Douglas-fir (*Pseudotsuga menziesii* var. *glauca* (Mirbel) Franco), Gambel's oak (*Quercus gambelli* Nutt), New Mexico locust (*Robinia neomexicana* A. Gray), and Silver-leaf oak (*Quercus hypoleucoides* A. Camus). *P. ponderosa* dominates AGB at the mid-range of elevations, with *P. strobiformis,* and *P. menziesii* increasing in proportion (<30%) as elevation increases.
2) ***Mixed-conifer forest***: Sites were located from 2,400 to 2,750 amsl with composition of >70% *P. menziesii* by volume. Primary species include Douglas-fir and white fir (*Abies concolor* (Gor. & Glend.) Lindl. ex Hildebr.), with minor components of Engelmann spruce (*Picea engelmannii* Parry ex. Engelm.), sub-alpine fir (*A. lasiocarpa* Hook.) Nutt.), ponderosa pine and southwestern white pine. Mixed-conifer sites are generally more mesic than pine forest PFTs. At upper elevations the species composition of northerly aspects of the mixed-conifer PFT shifts toward spruce and fir. At lower elevations and along southerly aspects, composition of the mixed-conifer PFT contains a larger proportion of pine and oak species.

3) ***White fir forest***: Sites were located between 2,200-2,750 amsl. With composition >70% *A. concolor* by volume. Sites were typically in ravines and steeply sided drainages in the Santa Catalina and Pinaleño, and along streams in the Valles Caldera. *P. menziesii* and *P. flexilis* trees were significant components. In the Santa Catalina and Pinaleño this PFT is where the largest *P. menziesii* trees were observed, suggesting this PFT is the most productive forest type in the study areas. This PFT has increased its distribution in the modern era as a result of fire suppression (O'Connor et al. 2014).

4) ***Spruce-fir forest***: The highest elevation PFT in the Pinaleño and Valles Caldera, occurs mainly above 2,900m amsl. The most common species in this type are *P. engelmannii* and *A. lasiocarpa*. The Santa Catalina have no endemic spruce; but do retain a small stand of corkbark fir (*A. lasiocarpa var. arizonica*). In the Pinaleño, recent large insect outbreaks and wildfires have severely damaged the extant spruce-fir forest PFT, with up to 83% of mature trees (DBH >7 cm) killed by insects in unburned areas, and 100% mortality in burned areas (Lynch 2009, O'Connor et al. 2014).

5) ***Aspen forest***: *Populus tremuloides* occurs between 2,100 and 3350 m and exists in early and late seral communities in all three locations. Aspen forest tends to occur on more mesic sites; it is found across the Valles Caldera at all elevations.

S2 Table. Vegetation Classification schemes common for all three study areas. The suggested biomass model for each classification scheme: Plant Functional Type (PFT, Smith *et al.* 1993), Existing Vegetation Type (EVT, Comer *et al.* 2003, Brohman and Bryant 2005, Muldavin *et al.* 2006, LANDFIRE 2010), USFS mid-scale dominance types (Mellin *et al.* 2008), Plant associations (Stuever and Hayden 1997), Potential Natural Vegetation Type (PNVT,

Nature Conservancy 2006, 2007), ReGap Analysis (Lowry *et al.* 2007, Prior-Magee *et al.* 2007), and Whittaker and Niering (1975)/Niering and Lowe (1984).

PFT Model	LANDFIRE EVT	USFS Mid-Scale Dominance Type	Plant Association	PNVT	Southwest ReGAP	W&N (1975) N&L (1984)
**Spruce-fir**	Inner-Mountain Basins Aspen-Mixed Conifer forest and woodland	Upper evergreen forest tree mix	Engelmann Spruce, Corkbark Fir Series	Spruce-fir forest	Rocky Mountain Subalpine Mesic Spruce-fir forest and woodland	Subalpine fir forest
**Mixed-Conifer**	Southern Rocky Mtn. Dry-Mesic Montane Mixed Conifer forest and woodland, Rocky Mtn. Montane Riparian	Upper evergreen forest tree mix	Douglas-fir and Limber Pine Series	Mixed conifer – frequent fire, Mixed conifer with aspen	Madrean Upper Montane Conifer-Oak Forest and Woodland, Rocky Mtn. Aspen forest and woodland	North-slope montane fir forest, Drier montane fir forest
**Ponderosa pine**	Madrean Lower Montane Pine-Oak forest and woodland, Madrean Pinyon-Juniper woodland	Ponderosa pine, Oak/Juniper/Pinyon mix, Upper Pine-Oak	Ponderosa pine Series, Apache Pine and Chihuahua Pine Series	Madrean Pine-Oak Woodland, Ponderosa pine-Evergreen Oak, Ponderosa pine forest	Madrean Lower Montane Pine-Oak Forest and Woodland, Madrean Pinyon-Juniper woodland, Madrean Encinal, Mogollon Chaparral	High-elevation pine forest, Low-elevation pine forest, Pine-oak forest, Pine-oak woodland
**White fir**		Upper evergreen forest tree mix	White Fir Series			Mesic ravine fir forest
**Aspen**				Mixed conifer with aspen	Rocky Mountain Aspen forest and woodland	Successional aspen

S1 and S2 References not in main text

Comer PD, et al. (2003) Ecological Systems of the United States: A Working Classification of U.S. Terrestrial Systems. NatureServe, Arlington, VA. 75 p.

LANDFIRE: LANDFIRE 1.1.0 Vegetation Dynamics Models. (2010) [Homepage of the LANDFIRE Project, U.S. Department of Agriculture, Forest Service; U.S. Department of the Interior] [Online]. Available: http://www.landfire.gov/index.php [2010, October 28].

Lowry R, et al. (2007) Mapping moderate-scale land cover over very large geographic areas within a collaborative framework: A case study of the Southwest Regional Gap Analysis Project (SWReGAP). *Journal of Remote Sensing of Environment, 108*: 59-73.

Lynch AM (2009) Spruce aphid, Elatobium abietinum (Walker): Life history and damage to Engelmann spruce in the Pinaleño Mountains, Arizona. In: Sanderson, H. Reed; Koprowski, John L., editors. The last refuge of the Mt. Graham red squirrel: Ecology of endangerment. Tucson, AZ: University of Arizona Press. p. 318-338.

Mellin T, Triepke FJ, Joria P. (2008) Mapping existing vegetation at the mid-scale level in the Forest Service Southwestern Region. In *Proceedings of the twelfth biennial USDA Forest Service remote sensing applications conference. Salt Lake City, Utah* (pp. 15-19).

Nature Conservancy (2006) Southwest Forest Assessment Project. Available at: http://azconservation.org/projects/southwest_forest_assessment

Nature Conservancy (2007) Historical Range of Variation for Potential Natural Vegetation Types of the Southwest. Available at: http://azconservation.org/downloads/historical_range_of_variation_for_potential_natural_vegetation_types

NatureServe (2005) International Ecological Classification Standard: Terrestrial Ecological Classifications. NatureServe Central Databases. Arlington, VA. U.S.A. Data current as of 19 October 2005.

Prior-Magee JS, et al., Editors. (2007) Southwest Regional Gap Analysis Project Final Report. U.S. Geological Survey, Gap Analysis Program, Moscow, ID. 422 pp.

Stuever, M, Hayden J. (1997) Plant Associations of Arizona and New Mexico, ed. 3, 7/1997. Vol 1: Forests, Vol 2: Woodlands (an update of the USDA Forest Serv SW Region Habitat Typing Guides. 9/1996, rev 7/1997. Contract R3-95-27).

White EP, Enquist BJ, Green JL (2008) On estimating the exponent of power-law frequency distributions. Ecology 89(4), 905-912.
