# Supplementary material for: Tree Morphologic Plasticity Explains Deviation from Metabolic Scaling Theory in Semi-Arid Conifer Forests, Southwestern USA
Source: PLoS One. 2016 Jul 8;11(7):e0157582. doi: 10.1371/journal.pone.0157582 (PMC4938440; doi:10.1371/journal.pone.0157582)
Supplement: S3 File — This file contains a workflow for classifying trees, a table of tree decay classes, and example plates of various tree species by class. (PDF) [file pone.0157582.s003.pdf]

Supporting Information 3: Tree Condition Codes

We use a character-assessment system during field work to assess tree condition in our field plots – for this paper we only used recently standing dead trees absent of damage to their apical leader. The following it the scoring rubric for determining individual tree condition.

1. Is the tree ***live or dead?***
Live (L)? Go to 2a.
Dead (D)? Go to 2b.

2a. Rate live tree macrocondition based on crown health, growth form, stem straightness and thickness, apparent vigor (see Live Tree Condition Scoring System below):
Excellent (LE)
Good (LG)
Fair (LF)
Poor (LP).

2b. Rate dead tree macrocondition:
Standing/leaning snag (DS*)
Fallen (DF*)
Stump or broken (DP*)
Then go to 3.

3. Dead tree decay class: choose the **first** of:
Foliage/fine branches present (D*F)
Bark remaining (D*B)
Main limbs remaining (D*L)
Bole intact (D*I)
Bole rotting, un-coreable (D*R)

Live Tree Condition Scoring System
Score 0-2 for each of the following five categorical variables, scoring "2" for trees in the best (healthiest) condition for that variable, "0" for trees in the worst (unhealthiest) condition. The minimum and maximum possible aggregate scores are 0 and 10 respectively. Condition class is determined by scores of:
1-3 Poor
4-6 Fair
7-8 Good
9-10 Excellent

Leaf condition. What is the condition of the leaves (needles)? Healthy trees have leaves or needles with rich, dark, saturated color (score 2). Unhealthy leaves are yellow (chlorotic) or even brown (dead or dying), which can reflect drought stress, insect attack, or nutrient starvation (score 0).

Leaf density. How much foliage is there in relation to the size and species of tree? Healthy trees have full canopies, needles well back along the branch (score 2). Unhealthy trees have few or sparse leaves or needles, often clustered at the tips (score 0).

Stem structural condition. Is the trunk of the tree structurally sound? Healthy trees have strong, straight upright stems, robust for their height and free from apparent injury (score 2). Unhealthy trees have weak, leaning, bent or crooked stems, appearing too weak for their height, or evidence of mechanical injury, lightning strikes (a characteristic spiral split often running the entire length of the tree)(score 0).

Insects, diseases, and parasites. Healthy trees are free from apparent signs of insect attack, parasites, or diseases. Unhealthy trees may show evidence of the following: Score 2 if there is no evidence of insects, diseases, or parasites, 1 if there is evidence of an attack that the tree was able to resist, and 0 if the tree appears affected by:

1. *Bark beetles* leave small (1-4 mm diameter or sometimes larger) circular or elliptical holes in the bark. A healthy tree can "pitch out" the boring insect, which may result in streams of dried pitch or sap running down the trunk.
2. *Other boring insects* may leave small piles of sawdust around the base of the tree as a symptomatic character.
3. *Leaf herbivores* (many of which are larval stages) result in leaves with chewed holes, blackened or yellowed portions, or other signs.
4. *Mistletoes* are hemiparasitic flowering vascular plants that attach to the host plant and derive its nutrition from the host. Pine mistletoes (*Artheucobium* sp.) are yellow-green in color and attach to branches or the main stem.

Apical meristem condition. Healthy trees have a healthy apical meristem, relatively few dead major branches, and evidence of steady stem elongation between annual nodes (score 2). The apical meristem of unhealthy trees may have been killed or injured by insects or lightning, resulting in a dead leader or forked tip. Unhealthy trees may also show little or no growth between years (although in old trees, vertical growth slows down naturally and is not necessarily a sign or poor health), and substantial dieback of the lower crown (as evidenced by many whorls of large dead branches, indicating the branch death is occurring faster than the natural shedding of lower branches).

S3 Table: Dead tree decay class If the tree is dead, then a decay class is recorded. The figures below and their corresponding descriptions are adapted from "Wildlife Habitats in Managed Forests of the Blue Mountains of Oregon and Washington" by Jack Ward Thomas, Agriculture Handbook No. 553, USDA Forest Service, September 1979.

Snag/Log Decay Code	Bark	Heartwood Decay	Sapwood Decay	Limbs	Top Breakage	Bole Form	Time Since Death
1	Tight, intact	Minor	None to incipient	Mostly Present	May be present	Intact	1-5 years
2	50% loose or missing	None to advanced	None to incipient	Small limbs missing	May be present	Intact	>5 years
3	75% missing	Incipient to advanced	None to 25%	Few remain	Approx. 1/3	Mostly intact	>5 years
4	75% missing	Incipient to advanced	25%+	Few remain	Approx. 1/3 to ½	Losing form, soft	>5 years
5	75%+ missing	Advanced to crumbly	50%+ advanced	Absent	Approx. ½+	Form mostly lost	>5 years

Plates

Examples of tree by condition class from our study

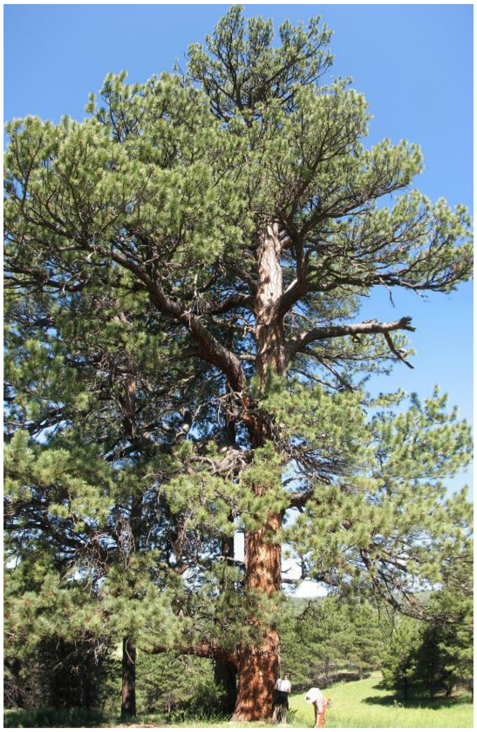

Plate 1. Example of an ‘excellent’ ponderosa pine, Valles Caldera. Photo Credit T. Swetnam.

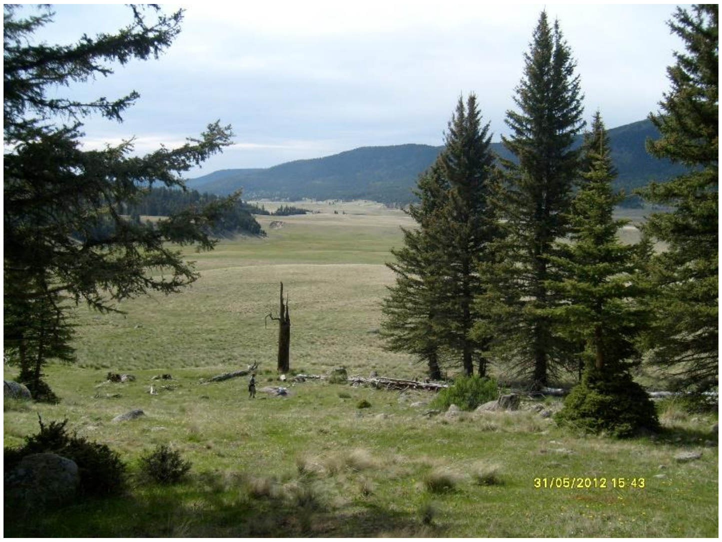

Plate 2. Example of ‘excellent’ and ‘good’ condition Engelmann spruce trees (right foreground), Valles Caldera. Photo Credit T. Swetnam.

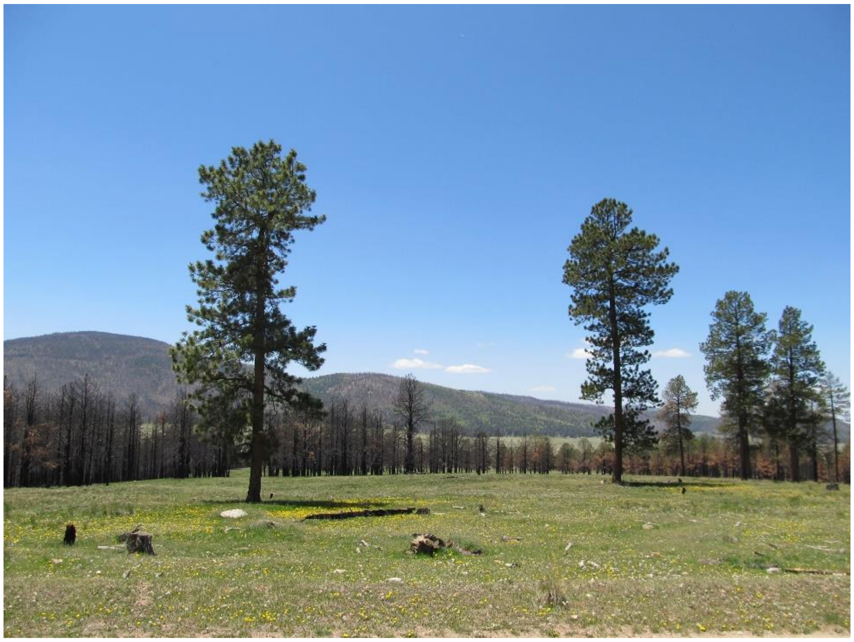

Plate 3. Examples of ‘good’ ponderosa pine (foreground), standing dead in background. Photo Credit C. O’Connor.

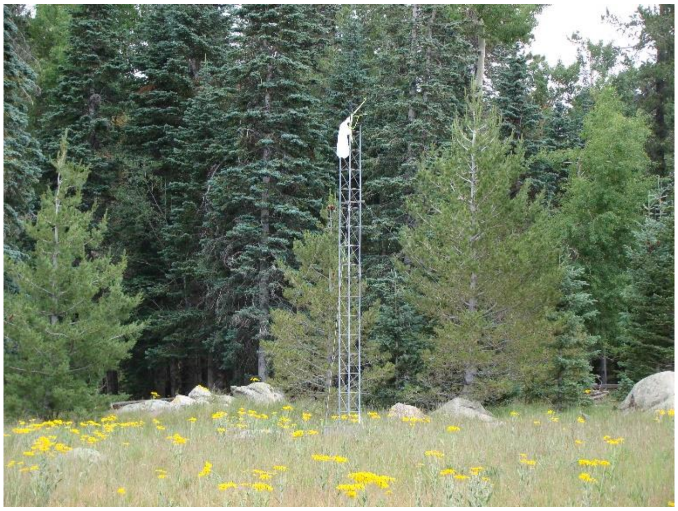

Plate 4. Examples of ‘good’ spruce and fir near Columbine Ranger Station, Pinaleño Mountains. Photo Credit C. O’Connor.

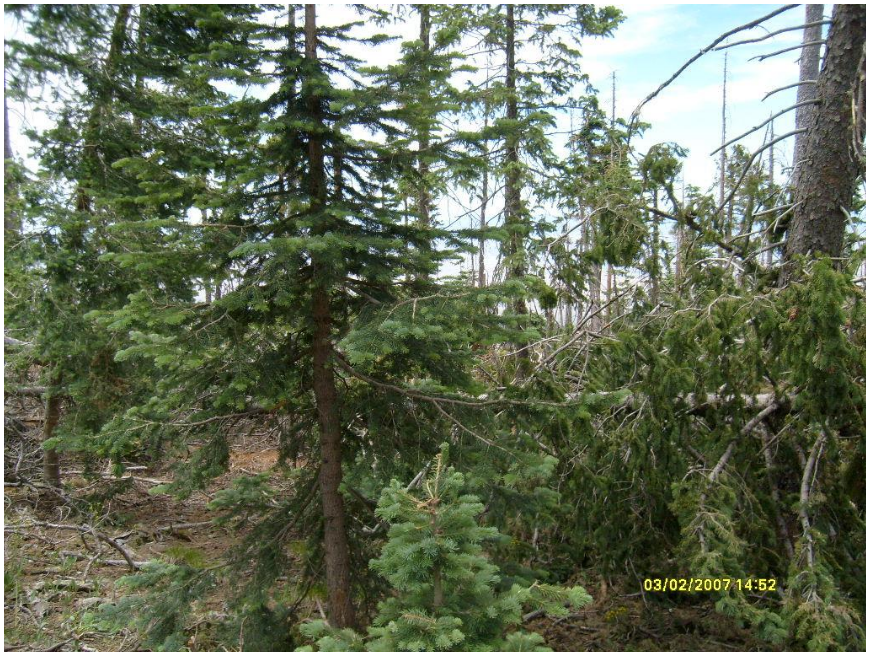

Plate 5. Example of ‘fair’ (foreground), ‘poor’ and standing dead (background) in spruce-fir, Pinaleño Mountains. Photo Credit C. O’Connor.

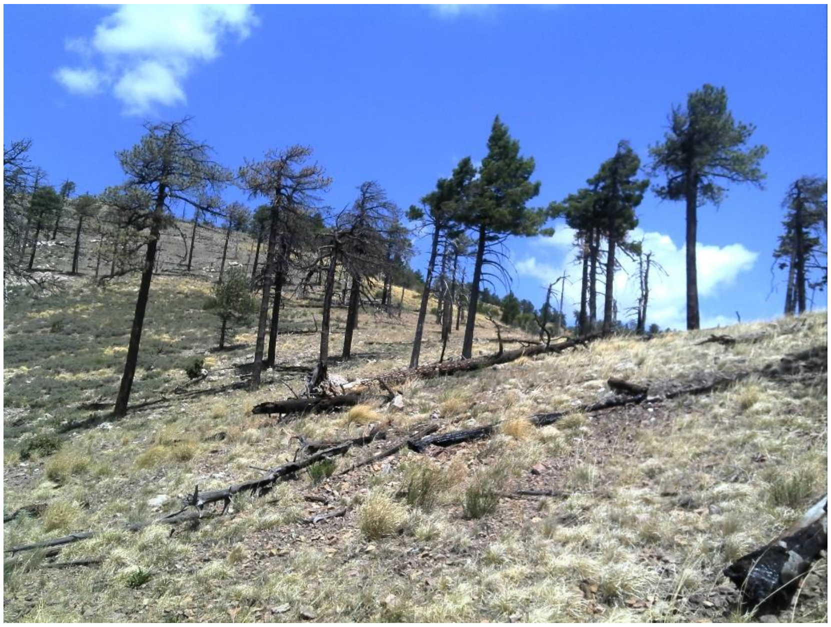

Plate 6.: Examples of ‘fair’ and standing dead in ponderosa pine. Photo Credit C. O’Connor.

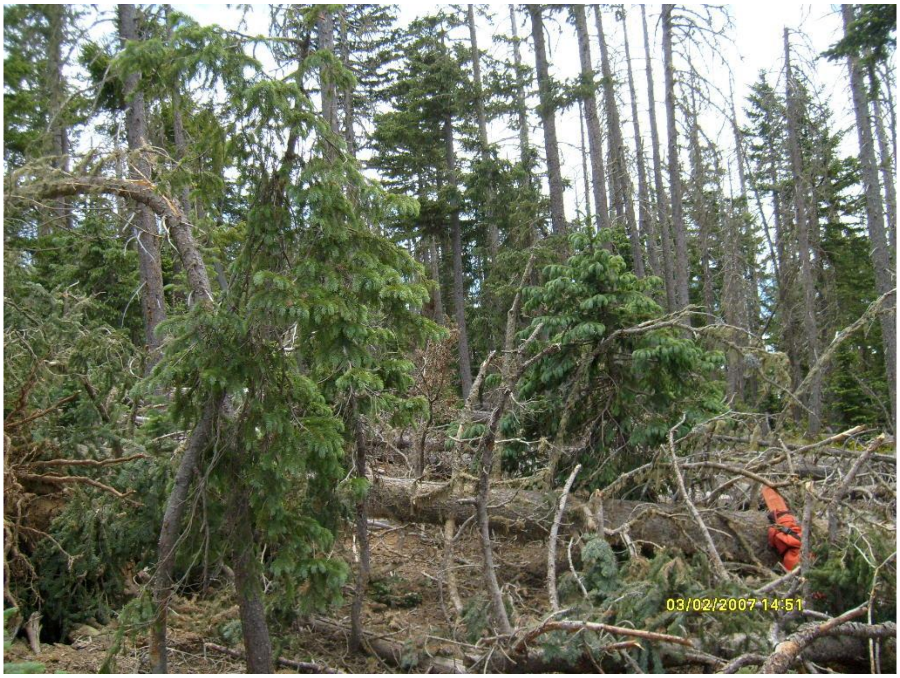

Plate 7. Examples of ‘poor’ and standing dead in spruce-fir, Pinaleño Mountains. Photo Credit C. O’Connor.

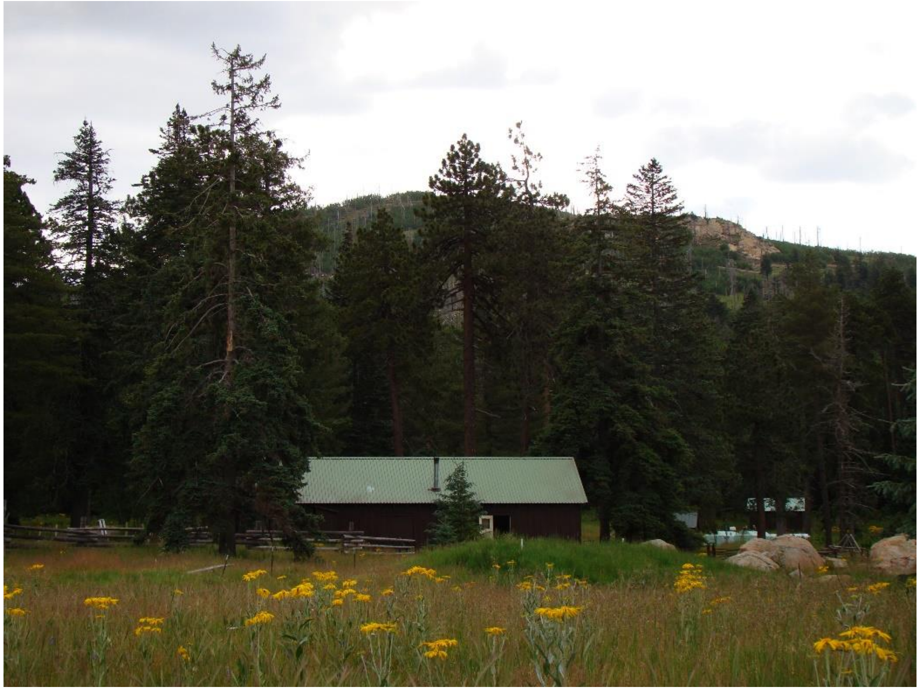

Plate 8. A ‘fair’ spruce in left foreground with loss of upper canopy and leader, Columbine Ranger Station, Pinaleño Mountains. Photo Credit C. O’Connor.

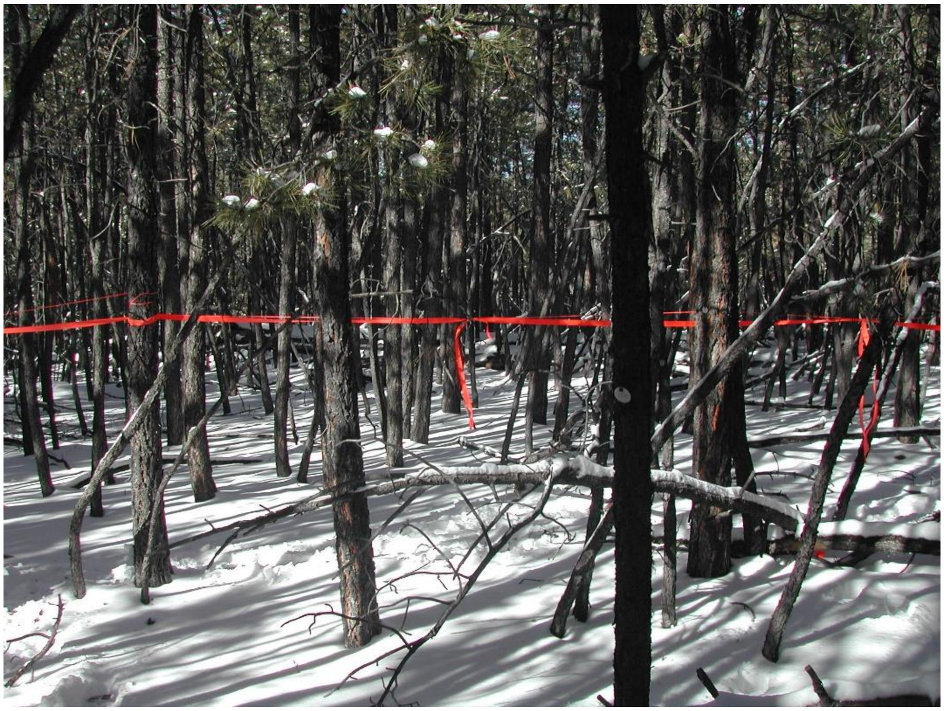

Plate 9. Doghair thicket of ponderosa pine in ‘fair’ and mostly ‘poor’ condition in Monument Canyon RNA. Photo Credit D.A. Falk.

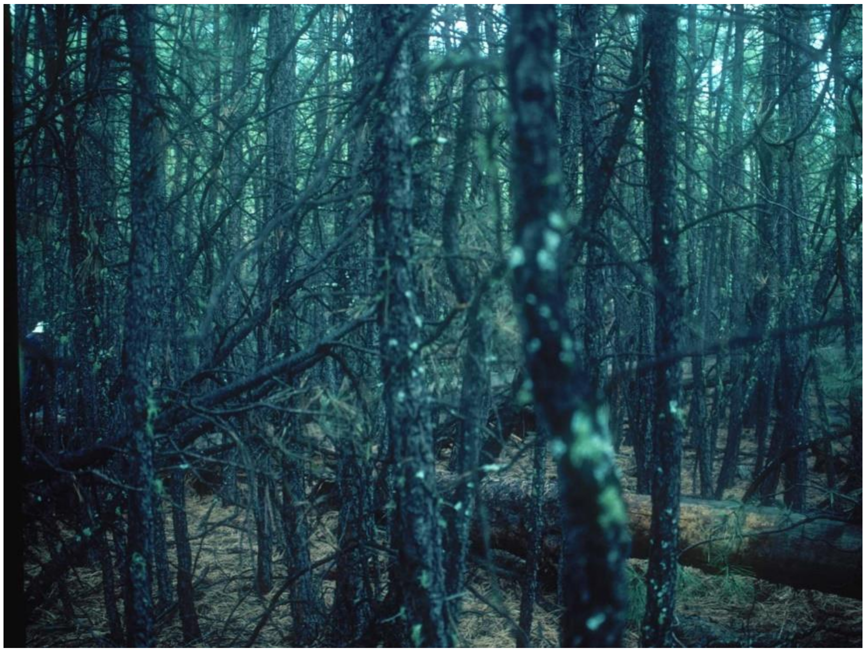

Plate 10. Doghair thicket of ponderosa pine in ‘poor’ and standing dead condition in Monument Canyon RNA. Photo Credit D.A. Falk.
