# Supplementary material for: Tree Morphologic Plasticity Explains Deviation from Metabolic Scaling Theory in Semi-Arid Conifer Forests, Southwestern USA
Source: PLoS One. 2016 Jul 8;11(7):e0157582. doi: 10.1371/journal.pone.0157582 (PMC4938440; doi:10.1371/journal.pone.0157582)
Supplement: S4 File — Scatter plots for the Pinaleño (n = 79, gold circles) and Valles Caldera (n = 48, blue circles) inventory plots are classified by: Canopy Cover percentage (CC%), EEMT (MJ m-2 yr-1), biomass M (Mg ha-1), Basal Area (m2), trees per hectare (t / ha-1), and plot average Tree Health (excellent = 4, good = 3, fair = 2, and poor = 1). A least-squares trend line is included for each study area. The Santa Catalina data are not shown. (PDF) [file pone.0157582.s004.pdf]

Supporting Information 4: Comparison between sites

In a comparison of biomass (Mg ha^{-1}), basal area (m^2 ha^{-1}), tree density (trees / ha^{-1}), and average tree health condition to canopy cover percentage (CC%) and productivity (as calculated by EEMT (Mj m^{-2} yr^{-1})), we found an increase in canopy cover was positively correlated with stem density, however there was considerable variation within cover percentage classes (S4 Fig 1). Increased canopy cover also tended to accompany increases in biomass and basal area (S4 Fig 1). In general, there was not a strong relationship between productivity and stand density or canopy cover percentage. However, in plots with greater productivity, maximum biomass and basal area also increased. Recent disturbances including wildfires are likely responsible for some plots having lower than expected biomass, basal area, and canopy cover, as was the case for several plots in the Spruce-fir from the Pinaleño.

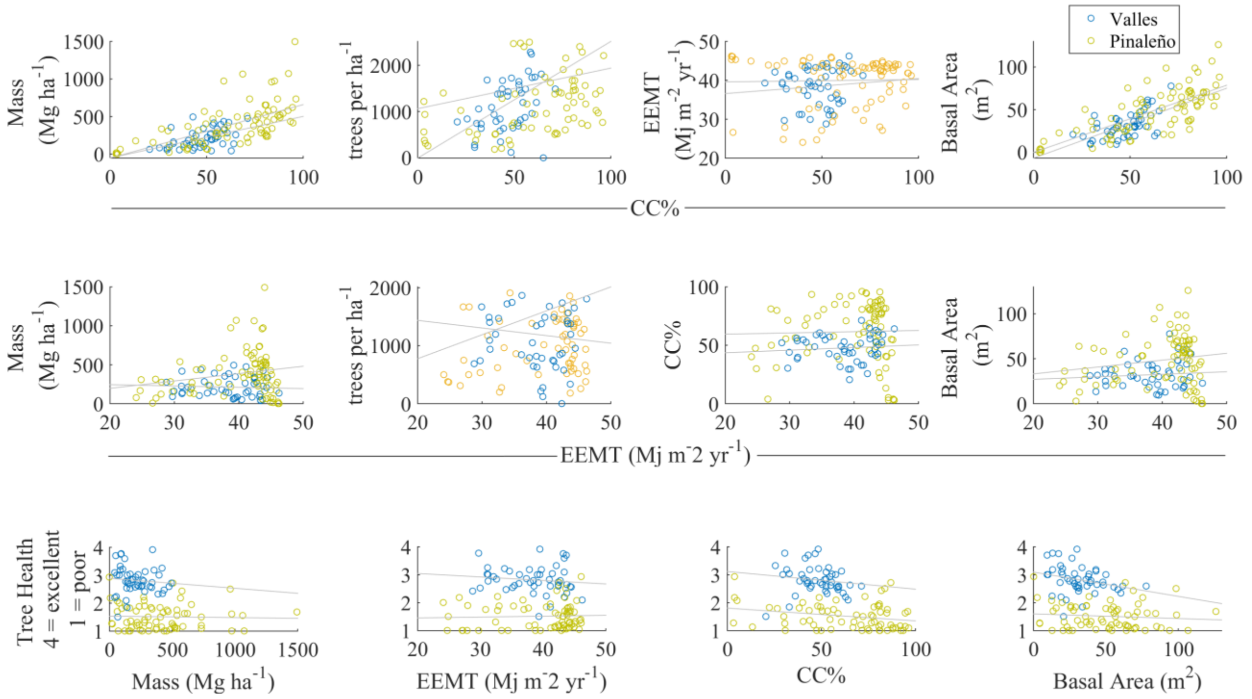

S4 Fig 1: Pairwise comparisons of plot metrics for the Pinaleño and Valles Caldera study sites. Scatter plots for the Pinaleño (n =79, gold circles) and Valles Caldera (n=48, blue circles)

inventory plots are classified by: Canopy Cover percentage (CC%), EEMT (MJ m^{-2} yr^{-1}), biomass M (Mg ha^{-1}), Basal Area (m^2), trees per hectare (t / ha^{-1}), and plot average Tree Health (excellent = 4, good =3 , fair =2 , and poor =1). A least-squares trend line is included for each study area. The Santa Catalina data are not shown.
